# Choking in Down Syndrome: Clinical and Forensic Perspectives

**DOI:** 10.7759/cureus.81046

**Published:** 2025-03-23

**Authors:** Pawan Kumar, Gokul G, Abilash S, Reisha Rijal, Devasenathipathy Kandasamy

**Affiliations:** 1 Forensic Medicine and Toxicology, All India Institute of Medical Sciences, New Delhi, New Delhi, IND; 2 Forensic Medicine and Toxicology, Vardhman Mahavir Medical College and Safdarjung Hospital, New Delhi, IND; 3 Radiodiagnosis and Interventional Radiology, All India Institute of Medical Sciences, New Delhi, New Delhi, IND

**Keywords:** airway obstruction, choking, down syndrome, forensic pathology, postmortem computed tomography (pmct)

## Abstract

Choking is a serious yet often overlooked risk among individuals with Down syndrome (DS), primarily due to their anatomical and neuromuscular vulnerabilities. This case report discusses the sudden choking-related death of an 11-year-old child with DS, emphasizing both clinical and forensic perspectives. The child developed respiratory distress while eating and became unresponsive, leading to a medicolegal investigation. A comprehensive postmortem examination, including verbal autopsy, postmortem computed tomography (PMCT), and traditional autopsy, was performed. PMCT revealed a foreign body obstructing the laryngeal inlet, along with pulmonary interstitial emphysema and diffuse pulmonary edema - findings that strongly suggested antemortem choking. The traditional autopsy also confirmed the presence of a soya chunk completely blocking the laryngeal inlet, with all findings aligning with the PMCT results. This case highlights the role of PMCT as a valuable, non-invasive tool in diagnosing choking-related deaths, especially in cases with minimal external indicators. Given that swallowing difficulties in DS persist into adulthood, early screening, caregiver education, and dietary modifications are critical preventive measures. Training caregivers to recognize choking risks, adopt safe feeding practices, and respond promptly to airway obstruction could significantly reduce such fatalities. Forensic pathologists should also consider choking as a differential diagnosis in sudden unexplained deaths, with PMCT aiding in a thorough assessment of airway compromise. Strengthening preventive strategies and improving diagnostic approaches can help mitigate the risk of choking-related deaths in individuals with DS.

## Introduction

Down syndrome (DS) results from having three copies of chromosome 21 (trisomy 21) in a few or all of the body’s cells. It is one of the most common genetic disorders, with prevalence ranging from one in 700 to one in 980 live births [1]. Cases of DS present with varying degrees of intellectual disabilities, dysmorphic features, airway abnormalities, vision problems, hypothyroidism, hypotonia, and congenital anomalies, which most commonly include congenital heart disease, followed by respiratory and gastrointestinal abnormalities [1-3]. Congenital heart anomalies affect at least half of DS cases, which include atrioventricular septal defects, persistent patent ductus arteriosus, and tetralogy of Fallot [4]. Gastrointestinal abnormalities can be due to structural abnormalities or problems digesting certain types of food [5,6]. Feeding difficulties are also seen in DS due to swallowing disorders involving the oral and pharyngeal phases caused by oropharyngeal sensorimotor function [7]. Though advances in medicine have improved the quality of life and life expectancy of children with DS, they are undoubtedly vulnerable to factors such as congenital heart defects, respiratory infections, and accidental choking [8]. Choking, defined as the obstruction of the upper respiratory tract, is a medical emergency where asphyxiation happens within minutes of the incident [9]. Choking is a significant cause of preventable mortality in individuals with DS due to hypotonicity of pharyngeal muscles, abnormal morphology of the oral cavity, and delayed initiation of the pharyngeal swallow reflex [10]. About 31-80 % of children with DS have drinking, eating, and swallowing difficulties, which persist till adulthood, with a prevalence of between 25% and 31% until the seventh or eighth decade [11]. Based on a cohort study, swallowing difficulties are six times more common in people with DS compared to people without DS [12]. Though children with DS have increased susceptibility to choking and aspiration, the clinical and forensic implications of the same are underreported and under-discussed. In forensic investigations, determining the cause of death in cases of suspected choking requires a meticulous approach. Postmortem computed tomography (PMCT) provides a non-invasive means of assessing airway obstruction, detecting foreign bodies, and evaluating associated pathological changes, thereby enhancing the accuracy and documentation of forensic examinations. We report a case of DS, in which the patient experienced a fatal episode of choking on a food bolus, and discuss the implications of the virtual autopsy with a focus on preventive measures to reduce the incidence of such outcomes.

## Case presentation

An 11-year-old female, who was a child with DS, was brought to the hospital in an unresponsive state. As per the parents, while she was having dinner at her residence along with other family members, she suddenly developed difficulty breathing and became unconscious. She was rushed to a nearby hospital immediately, where she was declared dead on arrival. The cause of death was not known; hence, a medicolegal case was registered, and the body was sent for postmortem examination. Postmortem examination was conducted within 24 hours of death at the Department of Forensic Medicine and Toxicology, All India Institute of Medical Sciences, New Delhi, India. As per the institute’s protocol, the postmortem examination included verbal autopsy, virtual autopsy using PMCT, and traditional autopsy.

Verbal autopsy revealed no previous choking/aspiration episodes and was unremarkable in terms of past medical history and family history, other than the presence of DS. The external examination revealed that the child was moderately nourished. External signs of DS such as short stature (<-3 SD for females according to IAP-2016/WHO-2005/CDC charts), increased upper-to-lower body ratio, short neck, increased intercanthal distance, short and broad hands, clinodactyly and widely spaced nipples were present. Bilateral conjunctivae were congested, and corneas were hazy. Bluish discoloration of the lips, oral mucosa, and all the fingernail beds were present.

PMCT was conducted using an Aquilion Lightning TSX-035A, a 16-slice multi-detector computed tomography (MDCT) spiral scanner (Toshiba America Medical Systems, Tustin, CA), with scan parameters of 120 KVp, auto mA, and exposure of 52 mAs. Image acquisition was performed with a slice thickness of 1 mm and a pitch factor of 0.8, utilizing a 16 × 1 mm collimation. The images were evaluated using standard window settings on Vitrea Software (version 7.10.1.20; Canon Medical Systems, Otawara, Japan).

**Figure 1:**
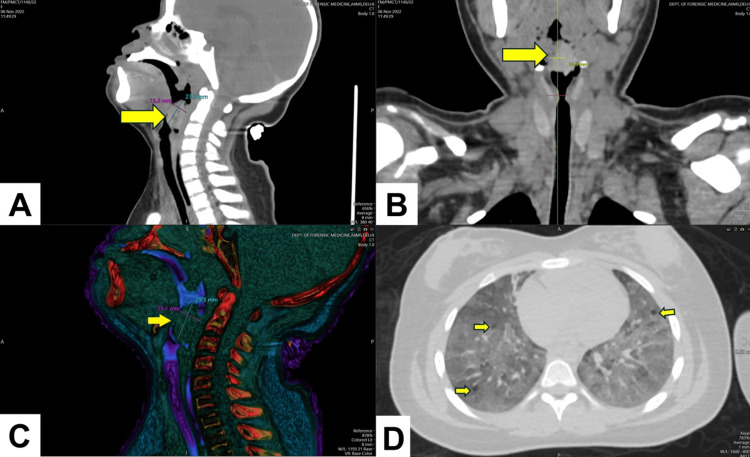
A-B: Multiplanar reconstructed (MPR) images showing mass (foreign body) at the level of the laryngeal inlet; C: Volume-rendered (VR) image showing the foreign body in relation to surrounding soft tissues; D: Axial section below the level of carina, showing pulmonary interstitial emphysema in all lobes of both lungs, suggestive of upper airway obstruction.

PMCT examination showed a heterogeneous mass/foreign body (HU= 87.1 ± 9.8) of size 2.75 cm x 1.52 cm at the laryngeal inlet without any attachment to the surrounding soft tissues (Figures 1A, 1B). The impaction of the mass/foreign object against the laryngeal inlet was also visualized with volume-rendered (VR) images (Figure 1C). Considering laryngeal obstruction due to mass/foreign body as the provisional diagnosis, other signs supporting and disputing the same were examined using PMCT. All lobes of both lungs showed pulmonary interstitial emphysema. The trachea showed minimal fluid collection; both lungs showed congestion with diffuse edema in all lobes (Figure 1D). The rest of the organs were unremarkable.

**Figure 2:**
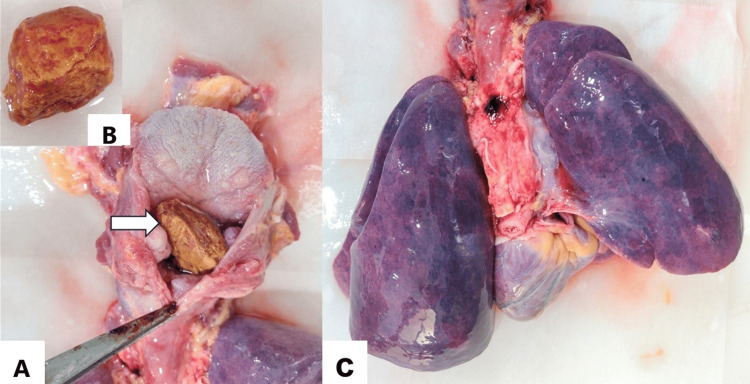
A: Image showing a chunk of soyabean impacted against the laryngeal inlet (arrow), with the inset image (B) showing the removed piece of soya chunk after dissection; C: Both lungs showing marked congestion.

A traditional autopsy was performed to correlate PMCT findings and determine the nature of the mass/foreign body. Internal examination of the neck revealed a soya chunk (textured vegetable protein chunk) of size 3 cm x 1.5 cm stuck at the laryngeal inlet, completely obstructing the laryngopharynx. (Figures 2A, 2B). Frothy fluid was present in the trachea, bronchus, and bronchioles. Both lungs were congested and edematous (Figure 2C). On the cut section, blood mixed with frothy fluid oozed out from all lobes. The weight of the right and left lungs were 390 g and 360 g, respectively. The brain was congested and edematous. The rest of the organs were unremarkable on gross examination. The cause of death was opined as "choking in a case of Down syndrome."

## Discussion

Choking, particularly in children with DS, is a significant health concern due to the anatomical and physiological challenges that predispose them to airway obstruction [10]. Their anatomical features include neuromuscular variations, dental anomalies such as tooth loss, and periodontal disease. Certain physiological or behavioral problems in DS, such as bruxism, can cause tooth attrition, malocclusion, or inadequate chewing of food, resulting in an increased risk of aspiration or choking [7]. In children with choking/aspiration, the common symptoms include paroxysmal cough, wheezing, and dyspnoea with/without stridor [13]. However, some events of choking go unwitnessed, where the individuals are found unconscious at the scene of occurrence. A high degree of suspicion should be employed in such cases by the emergency physicians and, in some unfortunate cases, by the forensic pathologists. In the current case, the child had dyspnoea, following which she became unresponsive, and she was brought to the hospital, where she was declared as brought dead. The history of the case gave a possible clue of choking with sudden onset of difficulty in breathing and loss of consciousness while having food in a child with DS. This prompted a detailed PMCT examination of the airways before the autopsy, where the level of obstruction was established.

Studies have shown that computed tomography (CT) is effective in identifying foreign objects in airways, assessing the extent of airway compromise, and visualizing associated pathological changes in the lungs [14]. In cases of choking, employing PMCT as a primary diagnostic tool allows forensic pathologists to comprehensively examine the extent of airway occlusion or compromise and its effect on the surrounding structures while preserving the integrity of the anatomical structures. These findings from PMCT can guide further targeted examinations or sampling, wherever necessary. The distress of the family members associated with traditional autopsy procedures can also be minimized by performing a virtual autopsy or a minimally invasive autopsy, as the case demands. This is especially important in communities where autopsy is not culturally or religiously accepted. PMCT can help handle these situations well while balancing the legal requirements and upholding the family members' sentiments. In the current case, PMCT revealed obstruction of the laryngeal inlet by a heterogeneous mass, which was suspected to be a food bolus, considering the history. Pulmonary interstitial emphysema (PIE), visualized in both lungs, is commonly seen in obstructive lung diseases and mechanical ventilation [15]. The contraction of the inspiratory muscles against a completely occluded laryngeal inlet generated a high negative pressure within the lung parenchyma, leading to overdistension and rupture of alveolar walls. This resulted in air seeping into the pulmonary interstitium and the perivascular spaces, ultimately reaching the subpleural space. The process was accompanied by pulmonary edema in both lobes, which is a well-documented phenomenon in upper airway obstruction. The spontaneous respiratory efforts in the setting of concurrent upper airway obstruction increased negative intrathoracic pressure, causing augmented venous return and pulmonary transudation, which further contributed to edema. Additionally, pulmonary vasoconstriction in response to hypoxia further worsens the air exchange [16]. Though PMCT-facilitated virtual autopsy was sufficient in determining the cause of death, a traditional autopsy was performed to corroborate these findings.

Choking incidents in individuals with DS are common in early childhood; the deceased, in our case, was 11 years old [17]. Smith et al. reported an observational study on patients with DS, in which 56.5% of their participants exhibited coughing during meal intake, indicating a possible risk of aspiration and resultant recurrent upper respiratory tract infections [11]. In our case, the child developed difficulty breathing without any cry for help or coughing, as reported by family members, suggesting the possibility of complete rather than partial airway obstruction (where signs such as coughing, talking, or breathing are usually observed). In most cases, children with DS have pharyngeal phase dysphagia, which commonly leads to choking or aspiration [10,11,18]. However, problems in all phases of swallowing could be present in cases of DS [7]. Besides the anatomical and neurological impairments attributed to DS, the impaired immune response consequent upon lymphopenia, reduced T cell proliferation, decreased neutrophil chemotaxis, and secondary immunodeficiency (related to nutritional/metabolic factors) can exacerbate the development of pneumonia in recurrent episodes of dysphagia [19]. Such cases, in addition to the current case, highlight the urgent need for education on feeding patterns in this community, especially with children having DS or other feeding difficulties who require extra attention [18]. From the perspective of forensic pathologists, PMCT can be a better diagnostic tool for investigating such cases, as it facilitates in situ evaluation of airways, which is of paramount importance in solving such cases.

## Conclusions

This case of a child with DS succumbing to choking on a soya chunk during dinner serves as a poignant reminder of the vulnerabilities in individuals with DS. The findings emphasize several forensic and preventive recommendations. Healthcare providers should implement routine assessments of swallowing and feeding capabilities in individuals with DS, particularly during early childhood, as early identification of feeding difficulties can significantly reduce the risk of choking/aspiration incidents. Moreover, caregiver education is essential in preventing and managing choking risks. Training programs should be organized for caregivers about safe feeding practices, recognizing signs of choking, administering emergency maneuvers, and ensuring effective responses to mitigate choking risks. Lastly, dietary guidelines should be established for individuals with DS to promote safe eating habits. Implementing these guidelines in healthcare settings can significantly reduce the incidence of such cases. Additionally, forensic pathologists should consider choking as a differential diagnosis in similar presentations and employ PMCT for a non-invasive assessment.
